# Supplementary material for: Quantitative Proteomic Analysis of Brassica Napus Reveals Intersections Between Nutrient Deficiency Responses
Source: Plant Cell Environ. 2024 Oct 24;48(2):1409–28. doi: 10.1111/pce.15216 (PMC11695800; doi:10.1111/pce.15216)
Supplement: Supplementary file 1 — Figure S1. Enriched molecular function GO terms. Dotplot representation of enriched molecular function GO terms for significantly changing proteins (q‐value < 0.05; Log2FC > 0.58 or <‐0.58) that are up‐ or downregulated in shoot (a) or root (b) under each nutrient deficiency (‐N, ‐P, ‐K or ‐S). The size of the dots represents the gene ratio (number of proteins in the conditions/total quantified proteins in the study). The colour code of the dots represents the log10 (p‐value). Only GO categories with p‐value < 0.01 and total count < 500 are included. [file PCE-48-1409-s003.pdf]

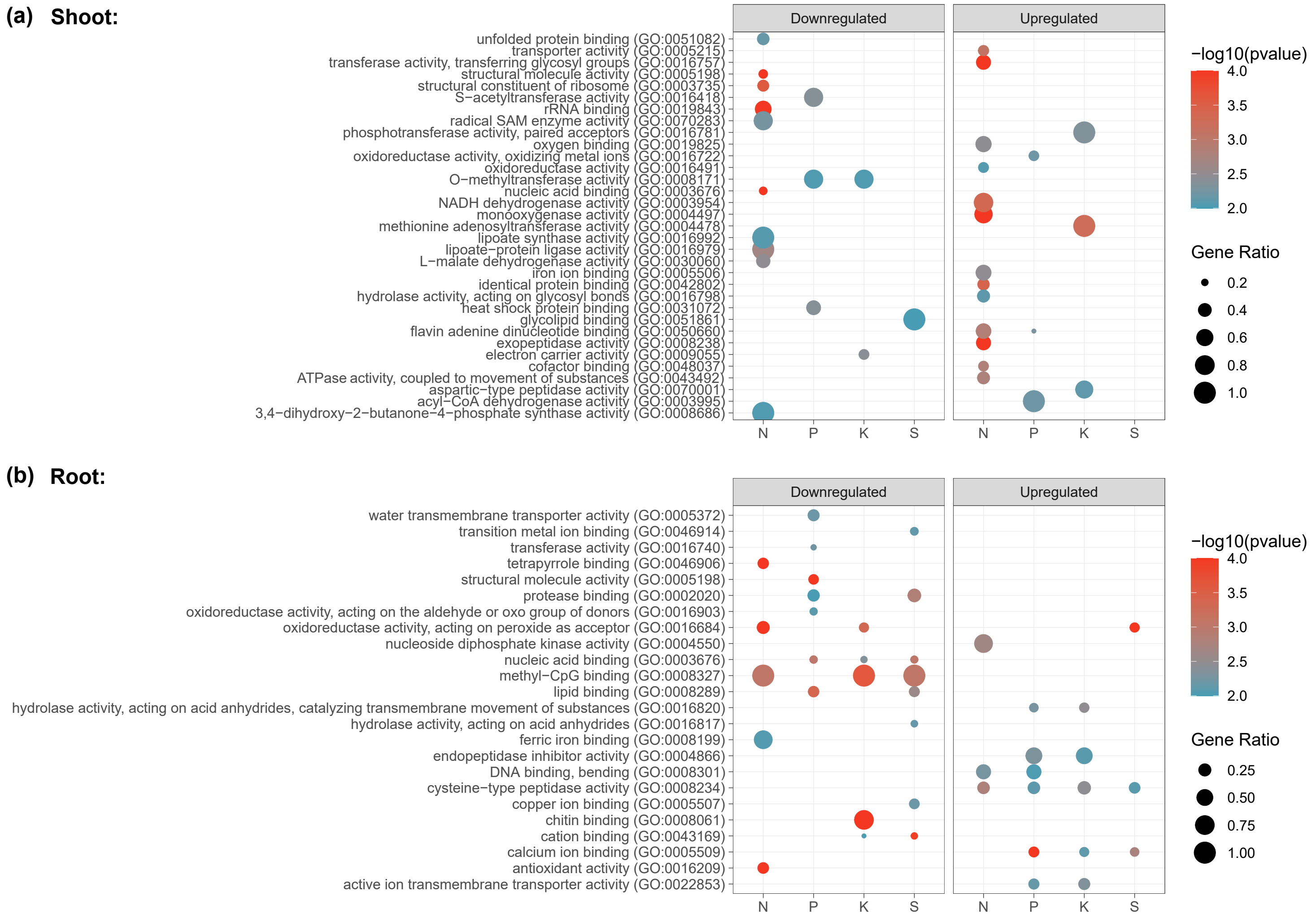

Figure S1. Enriched molecular function GO terms

Dotplot representation of enriched molecular function GO terms for significantly changing proteins (q-value <0.05; Log2FC >0.58 or <-0.58) that are up- or downregulated in shoot (A) or root (B) under each nutrient deficiency (-N, -P, -K or -S). The size of the dots represents the gene ratio (number of proteins in the conditions/total quantified proteins in the study). The colour code of the dots represents the log10(p-value). Only GO categories with p-value <0.01 and total count <500 are included.
