# Supplementary material for: Quantitative Proteomic Analysis of Brassica Napus Reveals Intersections Between Nutrient Deficiency Responses
Source: Plant Cell Environ. 2024 Oct 24;48(2):1409–28. doi: 10.1111/pce.15216 (PMC11695800; doi:10.1111/pce.15216)
Supplement: Supplementary file 2 — Figure S2. Root growth validation of four candidate proteins grown on media lacking nitrogen, phosphorus and sulphur. Root lengths were measured for wild‐type (Col‐0) and cml42, iqd32, chmp1b and hip1 loss‐of‐function seedlings 10 days following germination on N‐deficient, P‐deficient, and S‐deficient media. A non‐parametric Kruskal‐Wallis test, followed by uncorrected Dunn's test for multiple comparisons was used to determine statistical significance between nutrient‐deficient conditions and genetic background. Black violin plot indicates control conditions, purple indicates ‐N, pink indicates ‐P and yellow indicates ‐S. Letters denote statistical significance (p‐value < 0.05). Calmodulin‐like protein 42 (AT4G20780), cml42; IQ‐domain 32 (AT1G19870), iqd32; charged mutivesicular body protein 1B (AT1G17730), chmp1b; HSP70‐interacting protein 1 (AT4G22670), hip1. [file PCE-48-1409-s002.pdf]

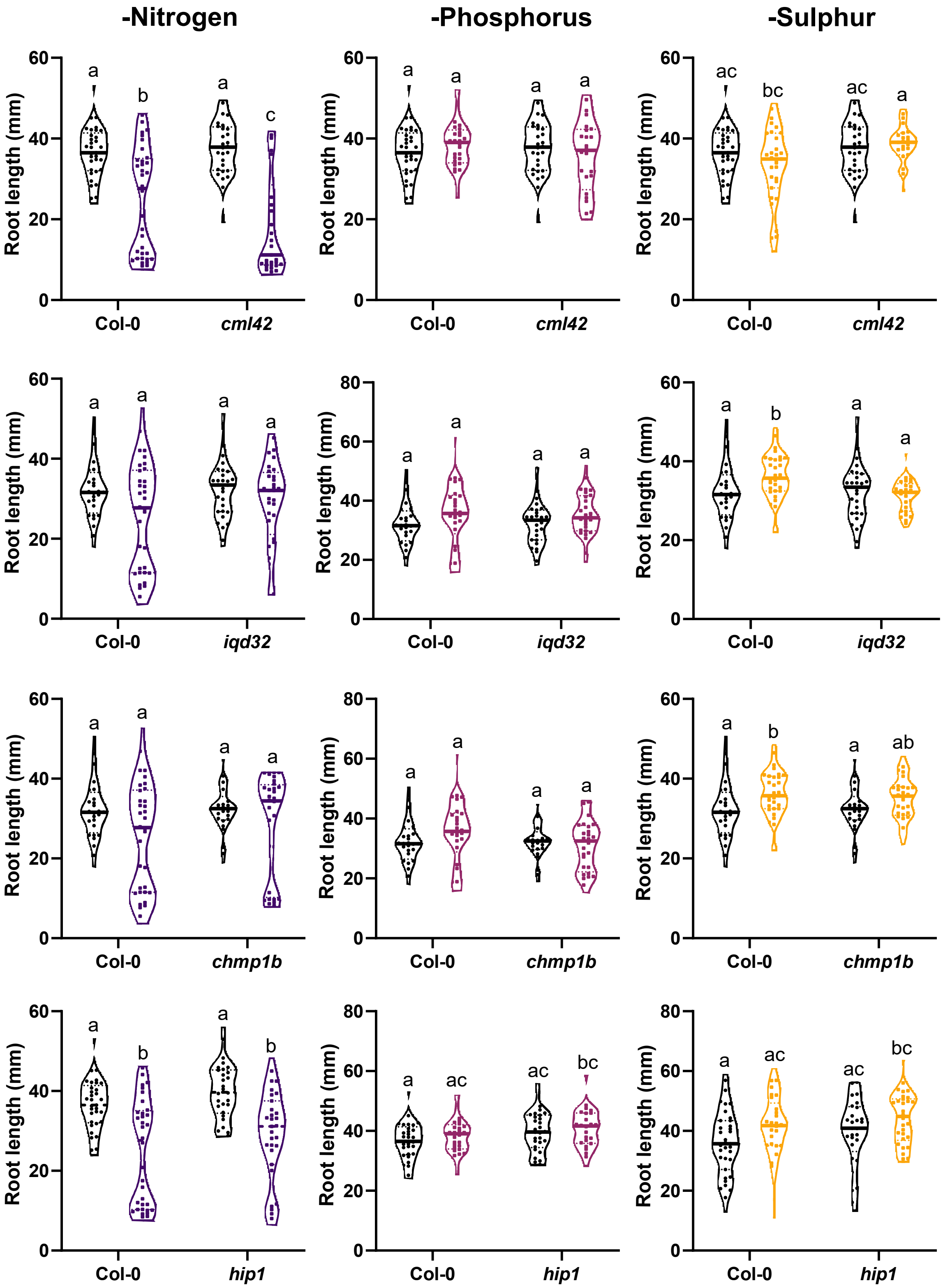

Figure S2. Root growth validation of four candidate proteins grown on media lacking nitrogen, phosphorus and sulphur Root lengths were measured for wild-type (Col-0) and *cml42*, *hip1, chmp1b* and *iqd32* loss-of-function seedlings 10 days following germination on nitrogen-deficient, phosphorus-deficient, and sulphur-deficient media. A non-parametric Kruskal-Wallis test, followed by uncorrected Dunn's test for multiple comparisons was used to determine statistical significance between nutrient-deficient conditions and genetic background. Black violin plot indicates control conditions, purple indicates -nitrogen, pink indicates -phosphorus and yellow indicates -sulphur. Violin plot middle bar indicates median value. Letters denote statistical significance (<0.05). *cml42*, calmodulin-like protein 42; *iqd32*, IQ-domain 32.
